# Norovirus, COVID-19, and Influenza Outbreaks Among Residents and Staff Members at the Eaton Wildfire Evacuation Shelter — Pasadena, California, January–February 2025

**DOI:** 10.15585/mmwr.mm7526a2

**Published:** 2026-07-09

**Authors:** Rudy Patrick, Katie Lee, Melody Kuan, Melany Chan, Sara Y. Tartof, Cameron Stainken, Shua J. Chai, Ellora Karmarkar, Matt Feaster

**Affiliations:** ^1^Pasadena Public Health Department, Pasadena, California; ^2^Kaiser Permanente Southern California, Pasadena, California; ^3^Epidemic Intelligence Service, CDC; ^4^California Department of Public Health, Richmond, California; ^5^Division of State and Local Readiness, Office of Readiness and Response, CDC.

**Summary**

**What is already known about this topic?**

Since 2015, multiple evacuation shelters have opened in response to California wildfires. High-density living conditions increase the risk for transmission of infectious diseases, increasing the risk for outbreaks in these shelters.

**What is added by this report?**

The Eaton wildfire evacuation shelter operated during January 7–February 16, 2025. Enhanced surveillance identified 104 cases of norovirus, 56 of COVID-19, 29 of influenza, and 30 of nonspecified respiratory illness. Implementation of isolation and infection prevention and control (IPC) protocols by multiple agencies were temporally associated with declines in reported cases.

**What are the implications for public health practice?**

Sustained and coordinated adherence to IPC measures in disaster evacuation shelters, particularly when multiple agencies are involved in relief operations, can protect residents and staff members from infectious diseases and mitigate outbreaks.

## Abstract

The Eaton wildfire burned during January 7–31, 2025, displacing approximately 100,000 residents, destroying 9,419 structures, and resulting in the deaths of 19 residents of the greater Pasadena, California, area. An evacuation shelter opened on January 7. On January 13, the Pasadena Public Health Department (PPHD) received reports of acute gastrointestinal illness, COVID-19, and influenza cases among shelter residents. An outbreak response was initiated, which included enhanced surveillance and improved infection prevention and control (IPC) measures. On January 18, additional assistance was requested from the California Department of Public Health (CDPH). During January 7–February 16 shelter operations, enhanced surveillance implemented in response to the outbreak identified 104 cases of norovirus, 56 of COVID-19, 29 of influenza, and 30 of nonspecified respiratory illness among residents and staff members. Reported norovirus, COVID-19, and influenza cases decreased sharply after January 22. The last case of reported illness was a COVID-19 case on February 6. Rapid implementation of isolation and IPC protocols and interagency communication were temporally associated with declines in reported cases. This response highlights the importance of ongoing adherence to and coordination of IPC measures for outbreak mitigation to protect the health of residents and staff members in shelters established in response to public health emergencies or disasters.

## Investigation and Results

The Eaton wildfire began on January 7, 2025, and was contained by January 31, 2025 (*1*). The fire burned 14,021 acres, destroyed 9,419 structures, and resulted in the deaths of 19 residents in the greater Pasadena area (i.e., Altadena, Pasadena, and Sierra Madre), an urban area with a population of approximately 230,000 persons (*1*,*2*).

### Opening of Wildfire Evacuation Shelter

Within the first 24 hours of the Eaton wildfire, approximately 100,000 residents were evacuated, including an estimated 1,800 residents of 25 assisted living facilities and 10 skilled nursing facilities (City of Pasadena, PPHD, unpublished data, 2025) and staff members and employees from PPHD and other City of Pasadena facilities. These estimates were derived using a count of facilities within the evacuation zone and licensed bed counts. An evacuation shelter was opened at the Pasadena Convention Center the same day the wildfire began. The convention center (approximately 97,000 sq ft) was divided into separate halls for the general population, families, residents with pets, and dining. Approximately 300 residents arrived on the first evening, January 7. By the following evening, the shelter housed approximately 1,700 evacuees.

### Implementation of Isolation and IPC Protocols

By January 11, PPHD had developed shelter isolation and IPC protocols for norovirus, COVID-19, and influenza based on local community wastewater trends, laboratory testing surveillance data,^†^ and documented infectious disease outbreak risks in evacuation shelters, including a norovirus outbreak during the 2018 Camp fire (*3*). On January 12, management of the Pasadena Convention Center, including IPC responsibilities, was transferred from PPHD to a nongovernmental organization (NGO) with experience operating emergency shelters as part of a disaster response protocol. During the transition, PPHD reduced on-site staff member presence and provided isolation and IPC protocols to NGO partners for outbreak preparedness. Clinical support was available on-site from health care providers throughout shelter operations.^§^ This report summarizes enhanced surveillance findings and the interagency IPC strategies implemented in response to concurrent norovirus, COVID-19, and influenza outbreaks in the shelter. This activity was reviewed by CDC, deemed not research, and conducted consistent with applicable federal law and CDC policy.^¶^

### Identification of Initial Cases 

On January 13, PPHD received reports from on-site health care providers of residents with positive COVID-19 and influenza test results and residents who were experiencing acute diarrhea or vomiting. In response, separate isolation halls were opened and patients with acute gastrointestinal illness (AGI) received testing. On January 18, after receiving additional reports of AGI and respiratory illnesses, PPHD requested assistance from CDPH.

### Enhanced Surveillance and Case Finding

PPHD and CDPH conducted enhanced surveillance by creating and validating line lists for cases of norovirus, COVID-19, influenza, and other nonspecified respiratory illnesses. Case counts were not mutually exclusive (i.e., persons could be included in more than one disease line list).

**Resident cases.** Resident cases were identified through passive care-seeking encounters with on-site medical providers. Systematic screening of asymptomatic persons was not conducted. Age, sex, reported symptoms, date of symptom onset, testing status (if available), date of testing and results, and hospitalization status were abstracted from on-site medical records. COVID-19 and influenza antiviral medications were not available on-site and treatment data during hospitalization were not collected.

**Staff member cases.** Cases among staff members were identified from on-site testing and illness reported to employers through passive reporting mechanisms; available data collected included onset date and symptoms. To protect staff member privacy and confidentiality, identifying information was not collected. By January 23, line lists were established for staff member cases from all on-site agencies, including cases retrospectively identified since commencement of shelter operations.

### Case Definitions

**Norovirus.** Persons with symptoms consistent with AGI were considered to have a probable case of norovirus. The first five identified patients with probable norovirus were offered a Biofire Diagnostics gastrointestinal panel.** Testing was discontinued after a norovirus outbreak was declared (at least two clinically compatible illnesses with a positive stool norovirus test result with the common exposure of being in the shelter); probable and confirmed (laboratory-positive stool sample) cases were subsequently considered together.

**COVID-19.** A confirmed case of COVID-19 was defined as a positive COVID-19 test result using a combined COVID-19/influenza rapid test (iHealth; Sunnyvale, California)^††^ in a person with signs and symptoms of COVID-19. These might include fever, chills, cough, shortness of breath, fatigue, sore throat, congestion, or runny nose.

**Influenza.** A confirmed case of influenza was defined as a positive influenza test result using the on-site combined COVID-19/influenza rapid test (iHealth, Sunnyvale, California) in a person with signs and symptoms of influenza. These might include fever, chills, cough, sore throat, body aches, fatigue, nasal congestion, or runny nose.

**Nonspecified respiratory illness.** A symptomatic respiratory illness in a person who either received a negative combined COVID-19/influenza rapid test result or who did not receive testing was considered a case of nonspecified respiratory illness. Nonspecified respiratory illness might reflect a heterogeneous mix of false-negative COVID-19 or influenza antigen test results, an infection with another respiratory pathogen, or a respiratory condition with a noninfectious cause. 

**FIGURE 1:**
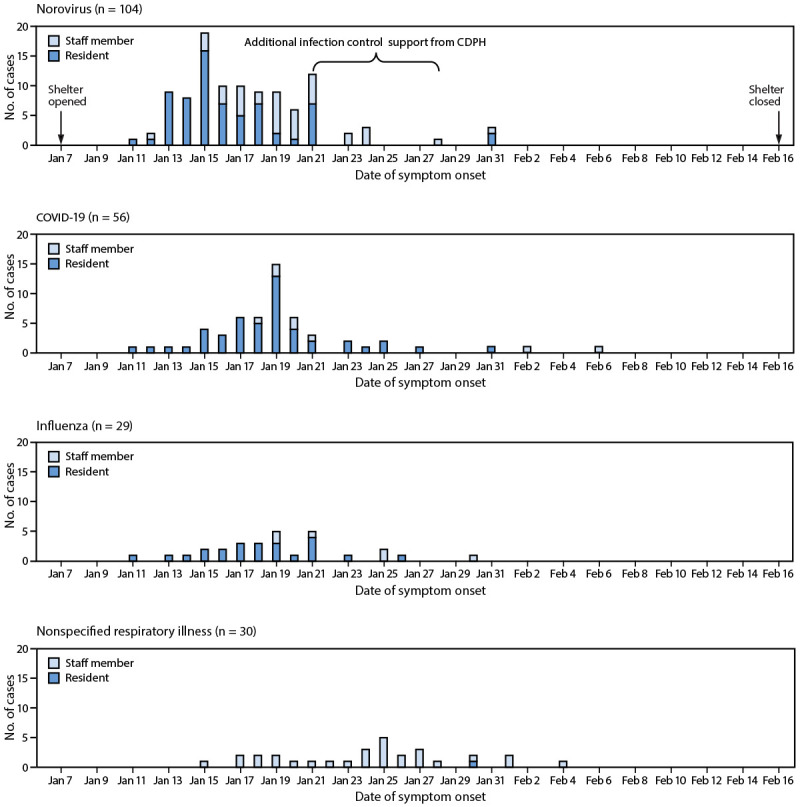
Number of confirmed and probable cases of norovirus,* COVID-19,^†^ influenza,^§^ and nonspecified respiratory illness^¶^ among residents and staff members of the Eaton wildfire evacuation shelter — Pasadena, California, January–February 2025 **Abbreviation**: CDPH = California Department of Public Health. * Norovirus cases include confirmed cases (laboratory-positive stool sample) and probable cases (symptoms consistent with acute gastrointestinal illness). ^†^ COVID-19 cases include confirmed cases (positive COVID-19 on-site combined COVID-19/influenza iHealth rapid test result) in a person with a clinically compatible illness. ^§^ Influenza cases include confirmed cases (positive influenza on-site combined COVID-19/influenza iHealth rapid test result) in a person with a clinically compatible illness. ^¶^ Nonspecified respiratory illness cases include no test result or negative COVID-19 and influenza test results in a person with symptomatic illness. ** Case counts for norovirus, COVID-19, influenza, and nonspecified respiratory illness are not mutually exclusive.

Shelter occupancy peaked during the first 24 hours with approximately 1,700 residents and decreased to approximately 200 residents during the last week of operations (February 10–16). Enhanced surveillance identified 104 norovirus (100 probable and four confirmed), 56 COVID-19, 29 influenza, and 30 nonspecified respiratory illness cases among residents and staff members (Table) (Figure 1). Eleven shelter residents had more than one diagnosis (four with norovirus and COVID-19, five with norovirus and influenza, and two with COVID-19 and influenza). Whether staff members also had multiple diagnoses could not be determined because staff member illnesses were reported as de-identified aggregate counts. Nine (8.7%) patients with norovirus infection and six (10.7%) with COVID-19 were hospitalized (Table).

**TABLE:** Cases of norovirus, COVID-19, influenza, and nonspecific respiratory illness identified among residents and staff members of the Eaton wildfire evacuation shelter — Pasadena, California, January–February 2025

Disease	No. (row %)*						First detection	Last detection
	Case status			Person with infection		Hospitalized		
	Total	Confirmed	Probable	Resident	Staff member			
Norovirus^†^	**104**	4 (3.8)	100 (96.2)^§^	66 (63.5)	38 (36.5)	9 (8.7)	Jan 11	Jan 31
COVID-19^¶^	**56**	56 (100.0)	0 (—)	48 (85.7)	8 (14.3)	6 (10.7)	Jan 11	Feb 6
Influenza**	**29**	29 (100.0)	0 (—)	23 (79.3)	6 (20.7)	0 (—)	Jan 11	Jan 30
Nonspecific respiratory illness^††^	**30**	—	—	1 (3.3)	29 (96.7)	0 (—)	Jan 15	Feb 4

* Case counts for norovirus, COVID-19, influenza, and nonspecified respiratory illness are not mutually exclusive.

^†^ Norovirus cases include confirmed cases (laboratory-positive stool sample) and probable cases (symptoms consistent with acute gastrointestinal illness).

^§^ Includes one resident who received a negative norovirus test result. After the norovirus outbreak definition was met (at least two illnesses with stool tests positive for norovirus), laboratory testing was discontinued, and persons with symptoms consistent with acute gastrointestinal illness (i.e., nausea, vomiting, or diarrhea) were considered to have a probable case of norovirus.

^¶^ COVID-19 cases include confirmed cases (positive COVID-19 on-site combined COVID-19/influenza iHealth rapid test result) in a person with a clinically compatible illness.

** Influenza cases include confirmed cases (positive influenza on-site combined COVID-19/influenza iHealth rapid test result) in a person with a clinically compatible illness.

^††^ Nonspecified respiratory illness cases include no test result or negative COVID-19 and influenza test results in a person with symptomatic illness.

## Public Health Response

**FIGURE 2:**
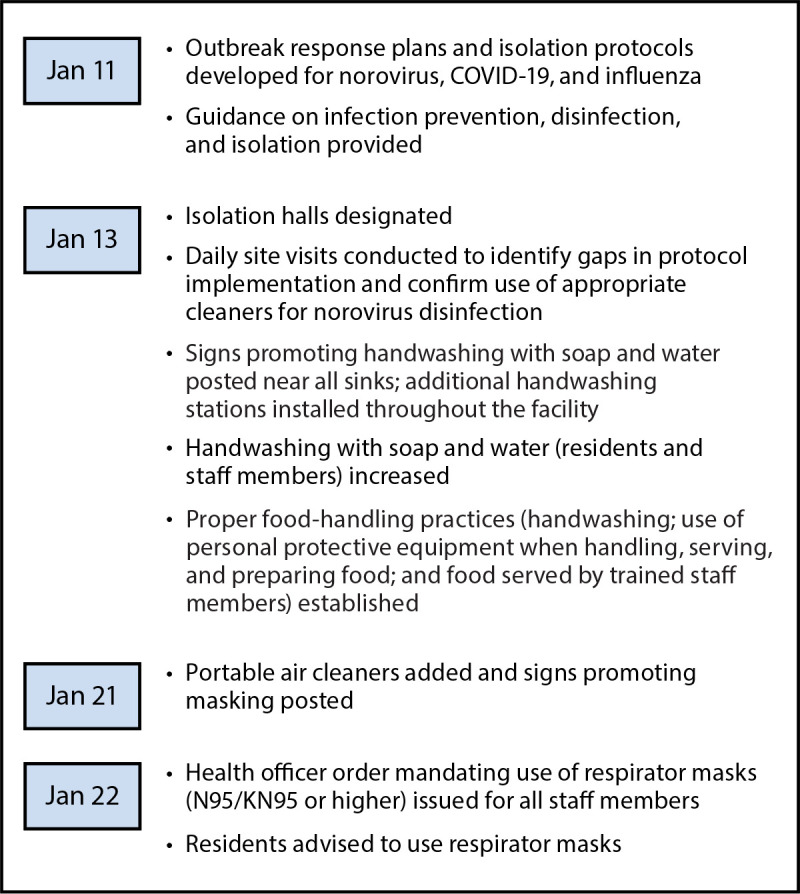
Implementation timeline for infection prevention and control interventions* at the Eaton wildfire evacuation shelter — Pasadena, California, January 7–February 16, 2025 * Persons with acute gastrointestinal illness, influenza, or nonspecified respiratory illness were isolated in separate areas (designated isolation halls) until 48 hours after symptoms subsided; persons in isolation had separate showers and bathrooms. Persons with COVID-19 were isolated in off-site hotel rooms for 10 days. Staff members were advised to wash hands with soap and water on entering and leaving isolation areas, and required to wear face masks. Use of recommended Environmental Protection Agency List G-approved cleaning disinfectants for norovirus was reinforced.

The first ill persons were identified on January 13; PPHD staff members conducted a site visit on the same day. Gaps in isolation protocol implementation were identified, including inadequate hand hygiene and personal protective equipment use and lack of an appropriate isolation area for ill persons separate from the general population. In addition, use of cleaners that were not effective against norovirus was observed; subsequently, recommended use of Environmental Protection Agency List G-approved disinfectants for norovirus was reinforced. After the site visit on January 13, additional IPC interventions were implemented by shelter staff members (Figure 2). Subsequently, PPHD and CDPH staff members conducted daily site visits and audits to ensure sustained IPC improvements and implementation. By January 27, after regular auditing and feedback regarding IPC, the presence of on-site cleaning personnel increased, recommended cleaning agents were used throughout the shelter, and adherence with respirator mask guidance was observed to be high among staff members. Reports of norovirus, COVID-19, and influenza cases decreased sharply after January 22. The last reported illness was a case of COVID-19 on February 6. All ill residents were released from isolation by February 12, and shelter operations ended on February 16 (Figure 1).

## Discussion

Infectious disease outbreaks in evacuation shelters are an important public health concern and exacerbate the challenges faced by persons seeking safety during and after an emergency (*4*). Outbreak control measures can be challenging to implement after disasters, when many persons with limited alternative housing options and varying medical needs are seeking immediate shelter (*4*). This challenge can be exacerbated when multiple communicable diseases are circulating within a community, each requiring a tailored prevention approach. Rapid implementation and sustained adherence to standardized IPC protocols should be part of standard shelter establishment procedures. Early adaptation of these measures can reduce transmission, morbidity, and mortality and decrease the likelihood of outbreaks. This report highlights the challenges of managing concurrent outbreaks with pathogen-specific transmission characteristics and control measures in a high-density shelter setting; however, because this analysis was observational, the effectiveness of specific interventions could not be definitively determined.

Implementing robust IPC measures before illness onset and hospitalization is a critical strategy for outbreak prevention and containment. To control outbreaks in the Eaton wildfire evacuation shelter, PPHD and CDPH partnered with NGOs and health care agencies to swiftly designate clear roles and responsibilities for IPC. PPHD and CDPH also performed regular IPC audits and enhanced surveillance to track illnesses among residents and staff members. This outbreak response underscored the value of establishing early and clear IPC assignments; ensuring the maintenance and transferability of IPC protocols throughout the entirety of shelter operations was critical to sustaining outbreak control efforts. Effective communication and collaboration among public health agents, shelter staff members, and health care partners supported improvements in surveillance and IPC implementation during concurrent outbreaks. Agreement to prioritize IPC implementation and standardize protocols for illness reporting and management among partners was crucial to maintaining consistent practices, even with staff member turnover. Extending surveillance and IPC measures to include staff members, while preserving confidentiality and privacy, enhanced illness identification and improved containment efforts. Regular audits and feedback regarding appropriate mask use, hand hygiene, and environmental cleaning practices, combined with interagency collaboration, helped maintain IPC standards and reduce transmission risk.

### Limitations

The findings in this report are subject to at least seven limitations. First, numbers of cases of COVID-19, influenza, and norovirus were likely underestimated because data were only available from persons who sought care; thus, milder cases might have been missed. Second, because of the low sensitivity of on-site COVID-19/influenza rapid tests, COVID-19 and influenza cases might have been misclassified as nonspecified respiratory illness. Third, surveillance of staff member illness was established later in the response, thus some cases might not have been retrospectively identified. Fourth, demographic characteristics could not be assessed for ill residents and staff members because information was not consistently collected. Fifth, trends in census at the shelter could not be assessed because movement of residents in and out of the shelter throughout the day and turnover of staff members prevented accurate daily census counts. Sixth, it was not possible to ascertain whether the declining shelter population over time contributed to fewer cases. Finally, information on antiviral treatment for COVID-19 and influenza was not collected because hospitalization and treatment data were not accessible.

### Implications for Public Health Practice

Interagency collaboration regarding the health and safety of shelter residents can support IPC of multiple potential outbreaks in evacuation shelters. This outbreak response highlights how developing early preparedness plans, establishing shared priorities among response partners (i.e., IPC and standardized disease surveillance), prioritizing communication among agencies during transitions in management, regularly identifying and improving gaps in shelter workflows (e.g., logistics, communication, and management), and ensuring consistent adherence to IPC measures are important to successful outbreak mitigation when multiple agencies are involved.

## Footnotes

* These authors contributed equally to this report

† Norovirus wastewater detections had been increasing in Los Angeles County since late November 2024, and the number of reported norovirus outbreaks in Pasadena was higher than in previous years (City of Pasadena, PPHD, unpublished data, 2025). The percentage of positive COVID-19 and influenza test results (3.5% and 26.1%, respectively) had been increasing in Los Angeles County in early January 2025.

§ Health care partners included Kaiser Permanente Southern California, AltaMed, International Medical Corps, and Los Angeles County Department of Mental Health. They provided on-site medical care, pharmacy services, and testing for COVID-19 and influenza.

¶ 45 C.F.R. part 46.102(l)(2), 21 C.F.R. part 56; 42 U.S.C. Sect. 241(d); 5 U.S.C. Sect. 552a; 44 U.S.C. Sect. 3501 et seq.

** Because PPHD was evacuated, AGI testing supplies were provided by Los Angeles County Public Health Department and testing was conducted by Los Angeles County Public Health Laboratory. Testing ceased after norovirus was confirmed.

†† Antigen tests for COVID-19 and influenza have low to moderate sensitivity, which might have resulted in misclassification.

## References

[1] California Department of Forestry and Fire Protection. Eaton fire. Sacramento, CA: California Department of Forestry and Fire Protection; 2025. https://www.fire.ca.gov/incidents/2025/1/7/eaton-fire

[2] US Census Bureau. 2018–2022 American Community Survey 5-year estimates. Washington, DC: US Census Bureau; 2023. https://www.census.gov/programs-surveys/acs/technical-documentation/table-and-geography-changes/2022/5-year.html

[3] Karmarkar E, Jain S, Higa J, Outbreak of norovirus illness among wildfire evacuation shelter populations—Butte and Glenn counties, California, November 2018. MMWR Morb Mortal Wkly Rep 2020;69:613–7. 10.15585/mmwr.mm6920a1 32437337 PMC7357343

[4] Yee EL, Palacio H, Atmar RL, Widespread outbreak of norovirus gastroenteritis among evacuees of Hurricane Katrina residing in a large “megashelter” in Houston, Texas: lessons learned for prevention. Clin Infect Dis 2007;44:1032–9. 10.1086/512195 17366445

